# Migration, Proliferation, and Differentiation of Cord Blood Mesenchymal Stromal Cells Treated with Histone Deacetylase Inhibitor Valproic Acid

**DOI:** 10.1155/2014/610495

**Published:** 2014-03-16

**Authors:** Leah A. Marquez-Curtis, Yuanyuan Qiu, April Xu, Anna Janowska-Wieczorek

**Affiliations:** ^1^Centre for Innovation (Formerly Research & Development), Canadian Blood Services, 8249-112 Street, Edmonton, AB, Canada T6G 2R8; ^2^Division of Hematology, Department of Medicine, University of Alberta, 8440-112 Street, Edmonton, AB, Canada T6G 2B7

## Abstract

Mesenchymal stromal cells (MSC) have great potential for cellular therapies as they can be directed to differentiate into certain lineages or to exert paracrine effects at sites of injury. The interactions between stromal cell-derived factor (SDF)-1 and its receptors CXCR4 and CXCR7 play pivotal roles in the migration of MSC to injured tissues. We evaluated whether a histone deacetylase inhibitor valproic acid (VPA) modulates the migration of cord blood (CB-) derived MSC towards SDF-1 and their proliferation and differentiation. We found that in MSC, VPA increased (i) the gene and total protein expression of CXCR4 and CXCR7 and primed migration towards a low gradient of SDF-1, (ii) the gene expression of MMP-2 and secretion and activation of proMMP-2, (iii) the proliferation and gene expression of pluripotency markers SOX2 and Oct-4, and exposure to lower concentrations of VPA (≤5 mM) had no effect on their differentiation to osteocytes and chondrocytes. Thus, our study indicates that VPA enhances the migration of CB MSC towards SDF-1 by increasing the expression of CXCR4, CXCR7, and MMP-2. VPA at low concentrations may be used for ex vivo treatment of MSC to increase their recruitment to sites of injury without compromising their ability to proliferate or differentiate.

## 1. Introduction

Mesenchymal stromal cells (MSC) have been shown to promote hematopoietic stem cell transplantation, alleviate graft-versus-host disease, treat disorders of the bone, cartilage, and muscle, and deliver therapeutic genes. The success of clinical applications of MSC relies upon the efficient recruitment and retention of these cells within the appropriate tissues. Although site-directed or local administration of MSC can result in successful engraftment, systemic infusion of MSC is still preferred as a minimally invasive mode of administration in majority of over 400 clinical trials currently listed on the U.S. National Institutes of Health website [1]. Thus, investigation of the mechanisms that regulate the migration and homing of MSC is crucial to the success of therapies utilizing MSC. Among mediators and receptors identified to provide migratory cues in MSC trafficking, the chemokine stromal cell-derived factor (SDF)-1 (also known as CXCL12) and its receptor CXCR4 have received considerable attention, and we have demonstrated that MSC migrate towards an SDF-1 gradient in vitro [2].

SDF-1 is upregulated at sites of injury and is considered a critical mediator of recruitment and migration of circulating CXCR4-expressing MSC, which are then able to stimulate structural and functional repairs in many organs. For example, it has been shown that SDF-1 protein is highly expressed in the periosteum of injured bone in a mouse model and promotes bone repair by recruiting intravenously transplanted MSC to the site of injury [3]. SDF-1 is also upregulated in the kidney of mice with renal ischemic/reperfusion injury, and MSC ameliorated this condition [4]. However, when administered systemically, only a small portion of the infused MSC home to the ischemic tissue, and the majority are entrapped in the lungs [5]. Therefore, in order to maximize the effectiveness of MSC-based therapies it is important to employ strategies that can enhance the recruitment and retention of infused MSC to their target tissues.

For most transplantation protocols, ex vivo expansion of MSC is necessary in order to attain a therapeutic dose. However, we and others have shown that the gene expression of CXCR4 declines with cell culture passage [2, 6] and that CXCR4 expression on the cell surface of MSC is low [7–9]. Previously, we reported that a histone deacetylase inhibitor (HDI) valproic acid (VPA) increases CXCR4 expression in CD34^+^ hematopoietic stem/progenitor cells (HSPC) derived from cord blood (CB) and their migration towards an SDF-1 gradient [10, 11].

HDIs are potential anticancer agents because of their abilities to alter gene expression, induce growth arrest and apoptosis of tumor cells, and stimulate differentiation [12]. VPA (2-propylpentanoic acid) is an anticonvulsant and mood-stabilizing drug approved by the Food and Drug Administration for the treatment of epilepsy and manic disorders [13]. It has been demonstrated that VPA elevated CXCR4 promoter-associated acetylated histone-H3 levels in rat MSC [14]. CXCR7 has been identified as another 7-transmembrane G protein-coupled receptor that recognizes SDF-1 as its ligand with an even greater affinity than CXCR4 [15]. Human bone marrow-derived MSC express the mRNA for CXCR7 and its knockdown decreases MSC migration [16]. Our present study was designed to investigate whether VPA enhances the expression of CXCR4 and CXCR7 in human CB MSC and their migration towards SDF-1. VPA has been shown to enhance proliferation and self-renewal of normal HSPC [17] and decrease multilineage differentiation potential of human MSC [18]. Here, we also investigated the effect of VPA on the self-renewal of CB MSC and their differentiation into osteogenic, chondrogenic, and myogenic lineages.

## 2. Materials and Methods

### 2.1. Cells and Cultures

CB was collected with the mother's informed consent in accordance with the guidelines of the University of Alberta Health Research Ethics Board. Light density mononuclear cells (MNC) were separated using Percoll (GE Healthcare Life Sciences, Baied'Urfe, QC, Canada) density gradient centrifugation and cultured in Iscove's modified Dulbecco's medium (IMDM; Invitrogen, Burlington, ON, Canada) supplemented with 10% fetal bovine serum (FBS, Invitrogen) as previously described by us [2]. For this study, 3 × 10^6^ MNC/cm^2^ was plated in T-25 tissue culture flasks and incubated at 37°C and 5% CO_2_, and after 24 h, nonadherent cells (including hematopoietic progenitors and mature blood cells) were removed and the complete medium replaced. The formation of an adherent layer was monitored closely and media changes were carried out every 5 days. As there is heterogeneity in the population of cells forming the adherent layer from various CB samples, only cultures containing a homogeneous population of cells showing fibroblastoid morphology were used (see Supplementary Figure 1(A) in the Supplementary Material available online at http://dx.doi.org/10.1155/2014/610495). Cells were passaged when they reached about 60% confluency. Several aliquots of passage 3 MSC were cryopreserved and later thawed and expanded. To avoid the high variability associated with populations of MSC obtained from different donors, MSC derived from one CB unit at passages 4 to 6 were used in all experiments. MSC were treated with or without (control) VPA (1, 5, or, 10 mM) for 3 or 6 h and used for subsequent experiments.

In order to confirm that the MSC used in all experiments satisfied the minimal requirements defining MSC [19], surface antigen expression was evaluated by flow cytometry using fluorescent-labeled antibodies CD34-PE and CD45-FITC (hematopoietic markers; Beckman Coulter, Mississauga, ON, Canada), CD73-PE, CD90-FITC, and CD105-PE (nonhematopoietic markers; BD Biosciences, Oakville, ON, Canada), and the appropriate IgG isotype controls. The MSC used in this study showed positive expression for the stromal markers CD73, CD90, and CD105 and negative expression for the hematopoietic markers CD45 and CD34 (Supplementary Figure 1(B)).

Further characterization of MSC involved their differentiation to various lineages. The cells were grown in Dulbecco's Modified Eagle Medium (DMEM) containing high glucose plus L-glutamine (Invitrogen) supplemented with 10% FBS and other components conducive to osteogenic, chondrogenic, or myogenic differentiation. Adipogenic differentiation was not carried out as CB-derived MSC have been shown to only poorly differentiate to adipocytes [20]. For osteogenic differentiation MSC were grown in media supplemented with 0.1 *μ*M dexamethasone, 0.2 mM ascorbic-2-phosphate, and 10 mM glycerol-2-phosphate (all from Sigma-Aldrich Canada, Oakville, ON, Canada). For chondrogenic differentiation MSC were grown in media containing 1 mM sodium pyruvate, 0.1 *μ*M dexamethasone, 0.2 mM ascorbic-2-phosphate, ITS+1 liquid media supplement (Sigma), and 10 ng/mL TGF-*β* (Peprotech, Rocky Hill, NJ, USA). For myogenic differentiation MSC were grown in media supplemented with 5% horse serum, 50 *μ*M hydrocortisone (Sigma), and 4 ng/mL basic fibroblast growth factor (Peprotech). At three weeks post-osteogenic and post-chondrogenic induction, cells were washed with phosphate buffered saline (PBS), fixed, and stained with Alizarin Red and Alcian Blue (both from Sigma) for detection of calcium deposits and proteoglycans, respectively. Cells were visualized and pictures were taken using an inverted light microscope and Micron software (Fisher Scientific, Nepean, ON, Canada).

### 2.2. Gel-Based and Quantitative Reverse Transcription-Polymerase Chain Reaction (RT-PCR)

Total ribonucleic acid (RNA) was extracted using TRIzol Reagent (Gibco-BRL, Gaithersburg, MD, USA) from untreated and VPA-treated MSC and from differentiated MSC according to the manufacturer's instructions. Complementary DNA was synthesized in a 20 *μ*L reaction volume containing 2 *μ*g total RNA and Moloney murine leukemia virus reverse transcriptase (Invitrogen) and used for RT-PCR. The human glyceraldehyde-3-phosphate dehydrogenase (GAPDH) and 18S RNA were quantified to normalize differences in the added RNA and efficiency of reverse transcription of gel-based and quantitative (q) RT-PCR, respectively. Primers for CXCR4, CXCR7, collagen X, myogenin, MyoD, osteocalcin, osterix, and GAPDH were obtained from Integrated DNA Technologies Inc. (San Diego, CA, USA), and the sequences and sizes of PCR products are listed in Table 1. Gels were visualized under ultraviolet light and densitometric analysis was carried out using the Fluorchem Imaging System (Alpha Innotech, San Leandro, CA, USA).

The other primers for qRT-PCR were obtained from Qiagen (QuantiTect Primer Assay, Qiagen, Santa Clarita, CA), namely, 18S (QT00199367), MMP-14/MT1-MMP (QT00001533), SOX-2 (QT00237601), and Oct-4/POU5F1 (QT00210840). Quantification was performed using Quantifast SYBR Green kit (Qiagen) in a 48-well plate on the StepOne Real-Time PCR system (Applied Biosystems, Foster City, CA, USA). The amplification cycle consisted of inactivation at 95°C for 5 min, amplification at 95°C for 10 s, and 60°C for 30 s and was run 40 times with each sample analyzed in triplicate. The level of target gene expression was determined using the comparative threshold cycle method (ΔΔ*C*_*T*_) and presented as fold-change of the target primer relative to the 18S RNA expression (StepOne Software v2.2.2, Applied Biosystems).

### 2.3. Flow Cytometric Analysis of CXCR4 and CXCR7

The surface expression of CXCR4 and CXCR7 on CB-derived MSC was examined using PE-conjugated antihuman CD184 (CXCR4, clone 12G5, BD Biosciences Mississauga, ON, Canada) and PE-conjugated antihuman CXCR7 (clone 11G8, R&D Systems, Minneapolis, MN, USA). Briefly, cells were washed three times in PBS containing 2% bovine growth serum and incubated with PE-labeled mouse IgG isotypic controls or CXCR4 and CXCR7 antibodies for 45 minutes at 4°C, followed by three washes. After the final wash, cells were fixed in 1% paraformaldehyde before flow cytometric analysis (FACscan; Becton Dickinson, San Jose, CA, USA).

### 2.4. Zymography and Western Blot

The gelatinolytic activities in serum-free media conditioned by untreated and VPA-treated MSC were assessed with the use of sodium dodecyl sulfate-polyacrylamide gel electrophoresis (SDS-PAGE). Briefly, MSC were incubated at 37°C in serum-free IMDM with or without (control) different concentrations of VPA (1, 2.5, 5, or 10 mM) for 3 or 6 h. After incubation, the cell-conditioned media were collected and analyzed under nonreducing conditions using 12% polyacrylamide copolymerized with 1.5 mg/mL gelatin (Sigma). The gels were washed with 2.5% Triton, incubated overnight in Tris-HCl buffer (pH 7.5) containing 5 mM CaCl_2_, and stained with 0.5% Coomassie brilliant blue G-250 (all from Sigma).

For Western blot, cell pellets were sonicated in lysis buffer (1% Triton, 10 mM Tris buffer, 150 mM NaCl, 1 mM EDTA, 1 mM EGTA, and pH 7.3) containing 1 mM phenylmethylsulfonyl fluoride and protease inhibitor cocktail (10 *μ*g/ml leupeptin, 10 *μ*g/mL aprotinin, and 1 *μ*g/mL pepstatin A, all from Sigma). Cell lysates were clarified by centrifugation at 14,000 rpm and 4°C for 10 minutes, and the protein concentrations were determined using the Bradford protein assay (Bio-Rad, Hercules, CA, USA). Samples were resolved by SDS-PAGE and transferred to a nitrocellulose membrane, followed by blockage with 5% fat-free dried milk in Tris buffered saline-Tween 20 for 1 h at room temperature. The membrane was probed with specific primary antibody as described for each experiment. Rabbit antihuman CXCR4 and CXCR7 antibodies were purchased from AbDSerotec (Raleigh, NC, USA) and GeneTex (Irvine, CA, USA), respectively. Mouse monoclonal antibody to *β*-actin (Abcam, Cambridge, MA, USA) was used for a loading control. After washing three times, the membrane was probed with HRP-conjugated specific secondary antibodies, and antibody binding was detected with ECL (SuperSignal West Pico system, Pierce, Rockford, IL, USA).

### 2.5. Chemoinvasion Assay

A chemoinvasion assay was performed as previously described [2]. Briefly, polycarbonate filters (13 mm diameter, 8 *μ*m pore size; Nucleopore, Toronto, ON, Canada) were coated with 25 *μ*g of Matrigel (Collaborative Biomedical Products, Bedford, MA, USA). The lower chamber contained IMDM supplemented with 0.5% bovine serum albumin only (control) or SDF-1 (20 or 100 ng/mL, Biomedical Research Centre, University of British Columbia, Vancouver, BC, Canada). Cells were preincubated with or without (control) VPA (5 mM) for 3 h at 37°C or with CXCR4 antagonist AMD3100 (Sigma) or active (CCX733) or inactivated (CCX226) CXCR7 antagonists (Chemocentryx, Mountain View, CA, USA). After incubation, cell medium was changed to fresh IMDM/0.5% BSA and cells were loaded into the upper chamber (1 × 10^5^ cells/chamber) and incubated at 37°C overnight. The next day, cells in the upper chamber were removed; the undersides of the filters were fixed with 4% formaldehyde and stained with 1% crystal violet. The migrated cells were counted under a light microscope by choosing three random fields.

### 2.6. Differentiation and Proliferation Assays

Cells were grown in DMEM supplemented with high glucose plus L-glutamine (Invitrogen), 10% FBS, and other components conducive to either osteogenic, chondrogenic, or myogenic differentiation as described above. Cells were pretreated with or without (control) VPA in serum-free IMDM for 3 or 6 h before switching to the differentiation media.

Proliferation assay was performed with the use of the LUMENESC-96 kit (a gift from Dr. Ivan Rich, HemoGenix Inc., Colorado Springs, CO, USA) which measures intracellular adenosine triphosphate (ATP) produced by the cells. Briefly, cells were cultured in a low serum medium (MSCGro, HemoGenix) in a 96-well plate (5000 cells/well, 6 replicates per condition) and incubated at 37°C and 5% CO_2_ overnight. The next day, cells were pretreated with or without (control) VPA (1, 5, or 10 mM) for 3 or 6 h. After treatment, VPA was washed out and cells were grown in MSCGro medium for another day. ATP standard curve and sample measurement were performed according to the manufacturer's instructions. Briefly, ATP Enumeration Reagent was dispensed into the wells and mixed to lyse the cells and release ATP, which acts as a limiting substrate of a luciferin/luciferase reaction. The bioluminescence emitted is measured using the SpectraMax L Luminometer (Molecular Devices/MDS Analytical Technologies, Sunnyvale, CA, USA). Data acquisition and analysis were carried out using the SoftMax Pro 5 software (Molecular Devices). The relative luminescence units were converted to standardized ATP concentrations after correcting for background luminescence contributed by the medium.

### 2.7. Statistical Analysis

Arithmetic means and standard deviations were calculated. Student's 2-tailed *t*-test was used to calculate *P* values and statistical significance was defined as *P* ≤ 0.05.

## 3. Results

### 3.1. Valproic Acid Increases the Gene and Protein Expression of CXCR4 and CXCR7 in CB MSC

To investigate whether CB MSC express the two SDF-1 receptors and to determine which receptor mediates SDF-1-directed MSC migration, mRNA was extracted from MSC, and qRT-PCR was performed using primers specific for CXCR4 and CXCR7. Because in rat MSC short-term (3 h) VPA treatment more robustly enhances CXCR4 transcript levels compared to long-term (24 h or longer) treatment [14], we exposed human CB-derived MSC to VPA for 3 or 6 h. As the plasma therapeutic level of VPA ranges from 0.35 mM to 1 mM, and VPA has been shown not to induce cytotoxic cell death in human adipose tissue-derived and umbilical CB-derived MSC after 24 h of treatment at doses less than 10 mM [18], we examined the effect of 1, 5, or 10 mM VPA on the gene expression of CXCR4 and CXCR7 in CB MSC.

**Figure 1:**
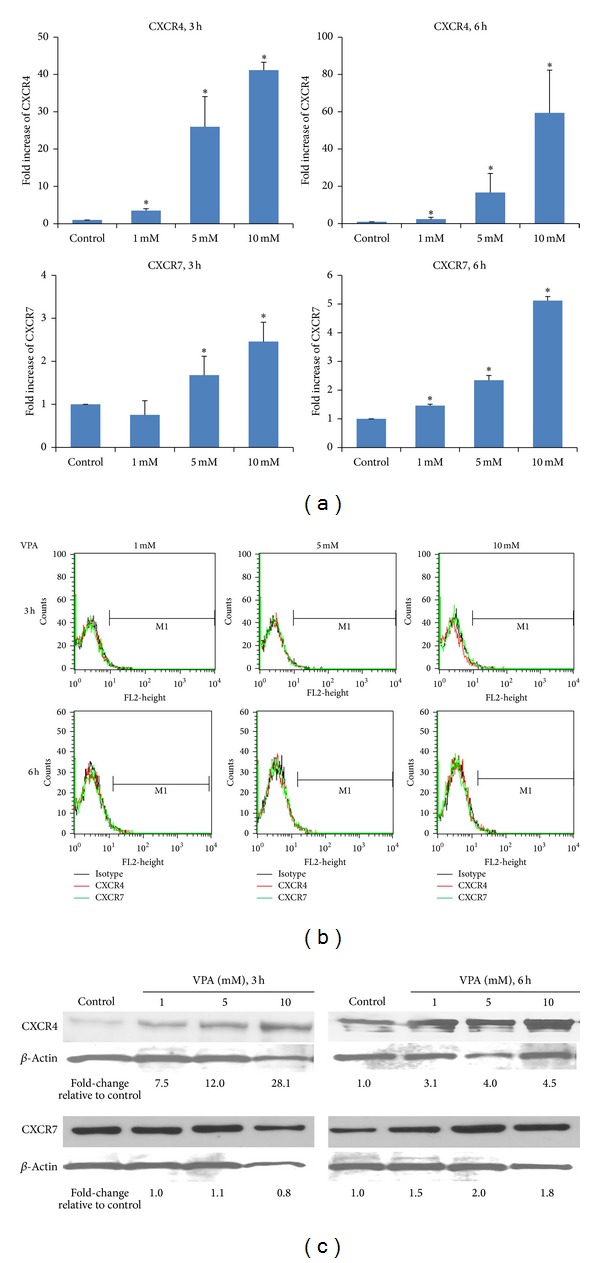
VPA increases CXCR4 and CXCR7 gene and protein expression. CB-derived MSC were treated with 0 (control), 1, 5, or 10 mM of VPA for 3 or 6 h. (a) Expression of CXCR4 and CXCR7 mRNAs was evaluated by real-time quantitative RT-PCR using 18S mRNA as internal calibrator. The data shown are based on two independent experiments. *P < 0.05 indicates statistically significant difference relative to control. (b) Surface expression of CXCR4 and CXCR7 was not affected by VPA treatment as determined by flow cytometry. The black line represents isotype control, the red line is for CXCR4, and the green line is for CXCR7. (c) Western blot of total CXCR4 and CXCR7 protein using *β*-actin as loading control. The numbers at the bottom of the gels represent the fold-increase in expression after VPA treatment relative to control. The data is representative of three independent experiments.

Both CXCR4 and CXCR7 mRNA were detected in MSC, and their expression was enhanced with increasing doses of VPA (Figure 1(a)). CXCR4 expression increased over 40-fold after 3 h and about 60-fold after 6 h of exposure to 10 mM VPA. The dose-dependent increase in CXCR7 gene expression was not as robust, just over 2-fold after 3 h and 5-fold after 6 h treatment with 10 mM VPA. To confirm whether the increase in the mRNA levels of CXCR4 and CXCR7 translated into an increase in protein expression, we examined cell surface and total protein expression in untreated and VPA-treated MSC, and, surprisingly, no upregulation was observed in the surface expression of either receptor after treatment with VPA (Figure 1(b)). It has been previously shown that CXCR4 is mostly sequestered intracellularly [21] and forms heterodimers with CXCR7 [22]. Therefore it is plausible that increase in protein expression may not be detectable on the surface of cells. However, using Western immunoblotting the expression of total CXCR4 and CXCR7 proteins was shown to be enhanced by VPA. A dose-dependent increase in CXCR4 levels was observed, reaching about 28-fold enhancement at 10 mM after 3 h. A longer incubation period (6 h) also showed a dose-dependent increase in CXCR4, but only by 4.5-fold with 10 mM VPA. Upregulation of CXCR7 protein expression reached a peak after 3 h and 6 h incubation with 5 mM VPA and started to decline at the highest (10 mM) VPA concentration used (Figure 1(c)).

### 3.2. VPA Enhances Migration of MSC towards a Low SDF-1 Gradient, Which Is Inhibited by CXCR4 and CXCR7 Antagonists

**Figure 2:**
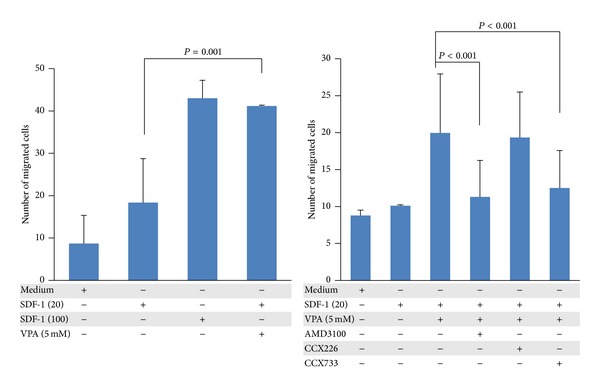
VPA enhances migration of CB MSC towards a low SDF-1 gradient which is inhibited by CXCR4 and CXCR7 antagonists. CB-derived MSC were preincubated with or without 5 mM VPA for 3 h and allowed to migrate across Matrigel towards a low (20 ng/mL) or high (100 ng/mL) SDF-1 gradient. Cells were also incubated with CXCR4 antagonist AMD3100, CXCR7 antagonist CCX733, or the inactive CXCR7 antagonist CCX226. The data are based on two independent experiments; *P* ≤ 0.001 indicates statistically significant difference.

To determine whether the increased CXCR4 and CXCR7 expression translates to an enhanced functional response, first we examined the in vitro trans-Matrigel migration of untreated cells. CB MSC migrated about 2-fold more towards a low SDF-1 gradient (20 ng/mL) and 4-fold more towards a high SDF-1 gradient (100 ng/mL) compared to medium alone (Figure 2, left panel). When the cells were pretreated with 5 mM VPA for 3 h before loading onto Boyden chambers, we found that VPA significantly increased (*P* = 0.001) trans-Matrigel chemoinvasion towards a low SDF-1 gradient to a level comparable to the chemoinvasion of untreated cells migrating towards a high SDF-1 gradient. This priming effect of VPA on chemoinvasion towards a low SDF-1 gradient was significantly (*P* < 0.001) inhibited by the potent specific CXCR4 antagonist AMD3100, supporting the hypothesis that the increase in chemoinvasion is due to a direct effect of VPA on CXCR4 expression. We also noted a significant (*P* < 0.001) inhibition of this priming effect by the CXCR7 antagonist CCX733, but not by the inactivated CXCR7 antagonist CCX226 (Figure 2, right panel).

### 3.3. VPA Increases MMP-2 but Not MT1-MMP Expression

**Figure 3:**
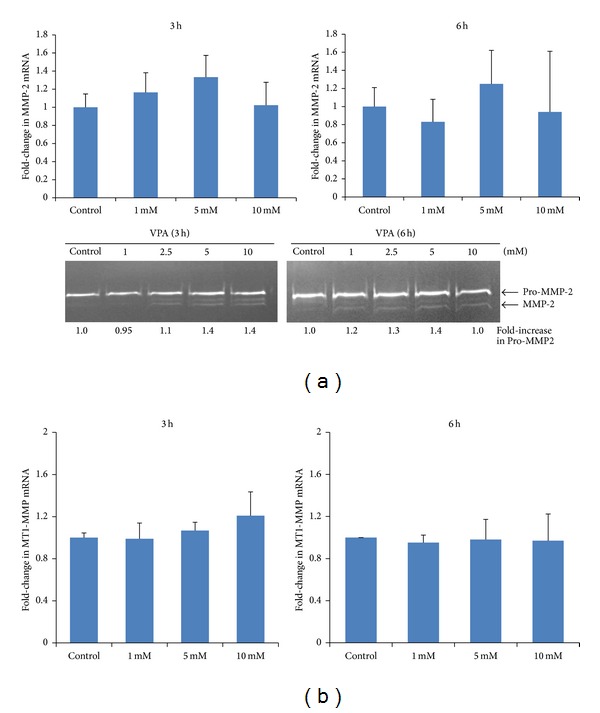
VPA increases MMP-2 but not MT1-MMP expression. CB-derived MSC were incubated with or without (control) 1, 2.5, 5, and 10 mM VPA for 3 h or 6 h. (a) The expression of MMP-2 mRNA (top panel) was evaluated by real-time quantitative RT-PCR (qRT-PCR) using 18S mRNA as internal calibrator. The secretion of pro-MMP-2 and active MMP-2 was examined using zymography (bottom panel). The numbers at the bottom of the gel correspond to the fold-increase in pro-MMP-2 expression relative to control. (b) The expression of MT1-MMP mRNA as determined by qRT-PCR was not affected by treatment of the cells with VPA.

Previously, we have shown that priming agents increase the homing-related responses of HSPC by upregulating their expression of basement membrane degrading enzymes matrix metalloproteinases (MMPs) [23]. We have also shown previously that MSC express MMP-2 and membrane type 1 (MT1)-MMP, but not MMP-9 [2]. Therefore, we examined whether VPA stimulates the expression of MMP-2 and MT1-MMP in CB MSC. The gene and protein expression of MMP-2 were detected using qRT-PCR and zymography, respectively.  Figure 3(a) (top left panel) shows that incubation of cells for 3 h with VPA resulted in a slight upregulation (1.3-fold) in the expression of MMP-2 mRNA which peaked at 5 mM VPA. Consistently, the secretion of proMMP-2 was enhanced 1.4-fold after 3 h of incubation with 5 mM VPA. VPA also induced activation of proMMP-2 as shown by the appearance of the truncated form of the enzyme (Figure 3(a), bottom left panel).  Figure 3(a) (top right panel) shows a slight upregulation (1.3-fold) in MMP-2 mRNA following 6 h of incubation with 5 mM VPA. However, a higher concentration of VPA (10 mM) caused an attenuation of proMMP-2 secretion (Figure 3(a), bottom right panel). MT1-MMP gene expression was not significantly affected by varying concentrations of VPA when CB MSC were incubated for 3 h (Figure 3(b), left panel) or 6 h (Figure 3(b), right panel).

### 3.4. VPA Exerts Different Effects on MSC Proliferation and Expression of Pluripotency Markers

**Figure 4:**
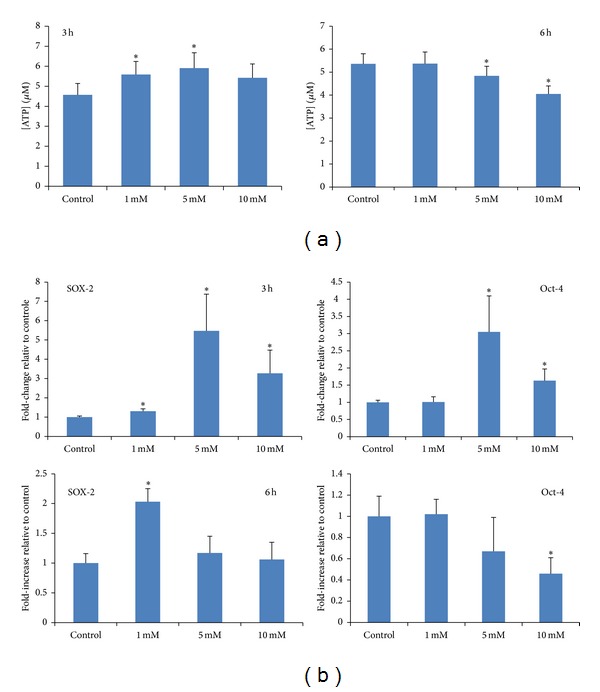
VPA exerts different effects on MSC proliferation and expression of pluripotency markers. (a) Cell proliferation was assayed by measuring the intracellular ATP released by CB-derived MSC after pretreatment with or without (control) 1, 5, or 10 mM VPA. The experiment was performed twice using 6 replicate wells per condition, and *P < 0.05 indicates statistically significant difference relative to control. (b) The expression of the pluripotency genes SOX2 and Oct-4 was evaluated by qRT-PCR using 18S mRNA as internal calibrator. The data is representative of two independent experiments and *P < 0.05 indicates statistically significant difference relative to control.

The effect of VPA on the proliferation of CB MSC was examined by measuring the amount of intracellular ATP released by viable cells. As shown in Figure 4(a), MSC proliferation significantly increased after a 3 h exposure to lower concentrations of VPA (1 mM and 5 mM). A higher concentration of VPA (10 mM) also elicited a slight, albeit not statistically significant, increase in cell proliferation. However, when the MSC were incubated with VPA (5 mM and 10 mM) for a longer period (6 h) a decrease in cell proliferation was observed.

As the pluripotency-associated genes SOX2 and Oct-4 are constitutively expressed by CB MSC, we examined the effect of VPA on the expression of these two genes using qRT-PCR. We found that following 3 h exposure to VPA, the expression of SOX2 and Oct-4 significantly increased and peaked at 5 mM VPA (over 5-fold and 3-fold, resp.) (Figure 4(b)). However, when MSC were incubated with high concentrations of VPA (5 mM and 10 mM) for a longer period (6 h), we found diminished expression of SOX2 and Oct-4 (Figure 4(b)). These results are consistent with the concentration- and time-dependent effects of VPA on cell proliferation.

### 3.5. Exposure of MSC to High VPA Concentration for a Longer Period Downregulates the Expression of Osteogenic, Chondrogenic, and Myogenic Markers

**Figure 5:**
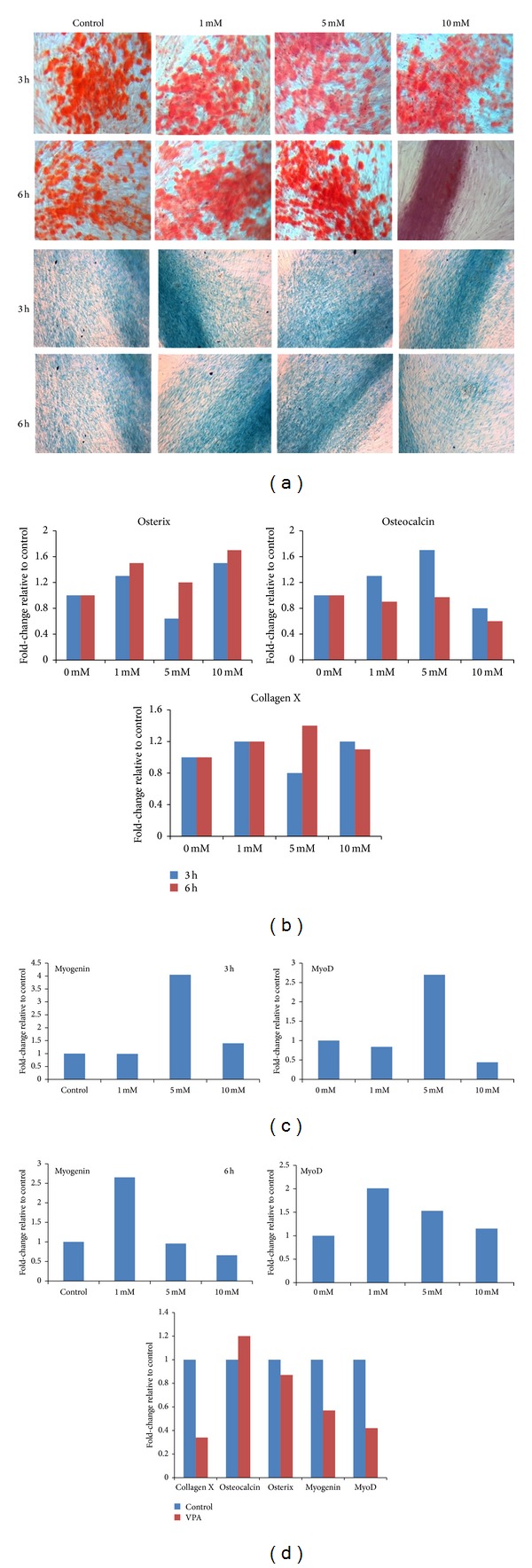
High VPA concentration for longer period downregulates the expression of osteogenic, chondrogenic, and myogenic markers. CB MSC were pretreated with or without (control) 1, 5, or 10 mM VPA for 3 h or 6 h. (a) The VPA was washed off and the cells were incubated in osteogenic and chondrogenic media. Differentiation into osteocytes was assessed after 3 weeks by staining for calcium deposits using Alizarin Red (top panel). Differentiation into chondrocytes was assessed after 3 weeks by staining for proteoglycans using Alcian Blue (bottom panel). (b) The expression of osteogenic markers (osterix and osteocalcin) and chondrogenic marker (collagen type X) was determined by gel-based RT-PCR using GAPDH as internal loading control. (c) The expression of myogenic markers (myogenin and MyoD) was evaluated by qRT-PCR using 18S as internal calibrator. The data is representative of two independent experiments. (d) CB-derived MSC were treated with or without (control) 5 mM VPA for 3 h and induced to differentiate the osteogenic, chondrogenic, and myogenic lineages. The gene expression of collagen type X, osteocalcin, osterix, myogenin, and MyoD was examined by gel-based RT-PCR using GAPDH as internal loading control.

The short-term exposure (3 to 6 h) of MSC to lower concentrations of VPA had no effect on their differentiation into osteocytes, as determined by Alizarin Red staining for calcium deposits, or into chondrocytes, as determined by Alcian Blue staining for proteoglycans (Figure 5(a)). However, there was a noticeable decrease in both osteogenic and chondrogenic differentiation when MSC were treated for 6 h with the highest concentration of VPA (10 mM).

We next examined by gel-based RT-PCR whether VPA affects the gene expression of osterix, a transcription factor that is essential for osteoblast differentiation and bone formation [24], osteocalcin, a protein secreted solely by osteoblasts that plays a role in bone mineralization and calcium ion homeostasis [25], and collagen type X, a protein produced by chondrocytes that is involved in the remodelling of cartilage [26]. Osterix, osteocalcin, and collagen type X are constitutively expressed by CB MSC, and VPA (1 and 10 mM) slightly increased the expression osterix after 3 and 6 h incubation (Figure 5(b)). VPA (1 and 5 mM) also slightly increased osteocalcin mRNA after 3 h but these concentrations had no effect after 6 h. However, it is worth noting that osteocalcin expression decreased when treated with 10 mM VPA. The expression of collagen type X slightly increased with 1 and 10 mM VPA after 3 h and after 6 h incubation with 5 mM VPA (Figure 5(b)).

We also examined the effect of VPA on gene expression of myogenin and MyoD, which are involved in the differentiation of primitive mesenchymal cells into skeletal muscle and are constitutively expressed by CB MSC. Using qRT-PCR, we found that VPA upregulates both myogenin and MyoD mRNA. After 3 h with 5 mM VPA, a 4-fold and 2.5-fold increase were observed in myogenin and MyoD; respectively, however, it took only 1 mM VPA to increase myogenin expression 2.5-fold and MyoD 2-fold when MSC were treated for 6 h (Figure 5(c)). On the other hand, a decrease in myogenin expression was observed when MSC were incubated with 10 mM VPA for 6 h; MyoD expression also decreased with 10 mM VPA after just 3 h (Figure 5(c)).

We then treated MSC with 5 mM VPA for 3 h, washed out the VPA, and induced the cells to differentiate to the osteogenic, chondrogenic, and myogenic lineages. After 3 weeks in the induction media, we examined the gene expression of osterix, osteocalcin, collagen X, myogenin, and MyoD in VPA-treated cells compared to untreated cells. We found that osteocalcin and osterix expression did not change significantly, whereas collagen type X, myogenin, and MyoD expression were diminished (Figure 5(d)).

## 4. Discussion

The ability of MSC to migrate to injured tissues is crucial to their applications in tissue repair and cellular therapy and is influenced by a wide range of chemokine-receptor axes, including SDF-1/CXCR4, and SDF-1/CXCR7. Modulating these interactions and directional signals could provide a potential way to enhance the recruitment of ex vivo-cultured MSC systemically infused to damaged or diseased tissues. Given that the expression of CXCR4 and other homing receptors is attenuated during culture expansion, the migration of MSC towards SDF-1 is diminished. Recently, we demonstrated that overexpression of CXCR4 with the use of cationic liposome-mediated transfection significantly increased MSC migration towards an SDF-1 gradient [9]. Here we show that short-term exposure of CB-derived MSC to VPA results in an upregulation in the gene and total protein expression of CXCR4 and CXCR7 and an enhanced chemotactic response towards SDF-1.

In spite of low CXCR4 or CXCR7 cell surface expression, MSC were responsive to the chemotactic signals from SDF-1, suggesting that in the presence of its own ligand, a proportion of intracellular CXCR4 or CXCR7 can be transposed to the cell surface. Previously, we showed that the initiator of the classical activation of the complement cascade (C1q) mediates the homing-related responses of HSPC [27]. C1q also enhances MSC migration by acting as a chemoattractant, which may facilitate their recruitment to inflammatory sites and potentiates their SDF-1-dependent migration by increasing the surface expression of CXCR4 [8]. There have been many attempts to enhance CXCR4 expression with the goal of stimulating chemotactic response to SDF-1 including treatment of MSC cultures with a cocktail of cytokines [28] or by lentiviral gene transfer to increase homing in vivo in irradiated hosts [29]. On the contrary, others have shown that lentiviral overexpression of CXCR4 and CXCR7 in murine MSC did not improve the homing and therapeutic potential of these cells in experimental acute kidney injury [30]. While both CXCR4 and CXCR7 are expressed by human MSC, only CXCR4 mediates their migration in response to tumor cells as shown in gene silencing experiments, and knockdown of CXCR7 had a minimal effect on migration of MSC [16]. Moreover, recruitment to ischemic kidney was increased in hypoxic-MSC compared to normoxic-MSC and this improved recruitment was abolished by neutralization of CXCR4 but not CXCR7 [4]. Although our study examined in vitro priming of MSC migratory responses by VPA, the ability of this HDI to promote MSC homing and to improve functional recovery was also assessed in vivo by other investigators using a rat model of cerebral ischemia [31]. MSC primed with VPA (2.5 mmol/L, 3 h) were transplanted into rats 24 h after transient middle cerebral artery occlusion (MCAO). Priming with VPA increased the number of MSCs homing to the cerebral infarcted regions. MCAO rats receiving VPA-primed MSCs showed markedly improved neurological score, reduced infarct volume, and increased microvessel density in the infarcted penumbra regions, and these beneficial effects of VPA priming were reversed by AMD3100 [31]. In the same study, lithium chloride also enhanced in vitro migration as mediated by MMP-9 [31].

The ability to cross the endothelial barrier and degrade the extracellular matrix is an essential step for MSC to reach target sites. We have previously shown that MT1-MMP and MMP-2 are involved in the migration of MSC [2] and that MMP-2 is enhanced during C1q-mediated MSC migration [8]. Here we show that VPA increases MMP-2 gene expression and the secretion and activation of proMMP-2. Our findings are consistent with other studies that demonstrate that inhibition of MMP-2 diminishes the transendothelial migration of MSC [32], while MMP-2 upregulation by inflammatory cytokines promotes their migration [33].

MSC, although not endowed with the hallmark characteristics of true stem cells, namely, pluripotency and self-renewal, still elicit clinical benefits by virtue of their capability to differentiate into adipocytes, osteoblasts, chondrocytes, myoblasts, and neuron-like cells. Because MSC undergo replicative cell senescence on repeated subcultures around passage 10 for our experiments, we used MSC no older than passage 6. Based on their epigenetic regulation of histones and DNA, HDI are able to induce differentiation and diminish self-renewal of stem cells. In this study, we investigated whether the HDI VPA affects the differentiation and proliferation of CB MSC. We found that the effect on cell proliferation was dependent upon the concentration of VPA used and the period of exposure of the cells. When MSC were treated for 3 h with VPA at concentrations of 5 mM or lower, proliferation was enhanced; however, longer incubation (6 h) with concentrations higher than 5 mM inhibited proliferation. This is consistent with the findings of others who showed that in a mouse osteoblast cell line VPA promoted cell proliferation at low concentrations (0.1 to 50 *μ*M for 3 days) and decreased cell proliferation and increased cytotoxicity at a high concentration (1 mM) [34]. Similarly, in mouse mesenchymal cells, proliferation was inhibited following a 48 h incubation with 6 mM VPA but otherwise had no effect relative to untreated cells [35]. The effect of VPA on cell proliferation was shown also to be dependent on the maturation level of the cells; immature cells were resistant to high concentrations of VPA [35]. Our results are in line with the report that VPA supplementation upregulates expression of pluripotency-associated genes, namely, Oct-4, Nanog, and SOX2 [36]. This is significant because HDIs are able to potentiate both stem cell differentiation and somatic cell reprogramming to pluripotency, and, in fact, replacement of oncogenic factors with small molecules such as VPA to improve the efficiency of reprogramming was recently described [37]. In particular, 2 mM VPA treatment for 1 week improved reprogramming efficiency by more than 100-fold, using Oct4-GFP as a reporter. VPA also enabled efficient induction of pluripotent stem cells without introduction of the oncogene c-Myc. VPA was the most potent of three HDIs tested (including suberoylanilidehydroxamic acid and trichostatin A), and it did not cause genetic changes when examined at the level of chromosomal abnormalities [38].

The underlying therapeutic mechanisms of MSC include not only their homing efficiency to injury sites, but also their ability to secrete trophic factors [39] and to differentiate into a variety of cell types. Constitutive expression by CB MSC of osteocalcin and osterix, collagen type X, MyoD, and myogenin, markers for osteogenic, chondrogenic, and skeletal muscle differentiation, respectively, implies that CB MSC cultures contain a subpopulation of cells that are capable of differentiating into these lineages. We observed a transient increase in the expression of these markers during short-term incubation (3 h) with low concentrations of VPA (<5 mM). However, we found a decrease in the expression of osteocalcin, collagen type X, myogenin, and MyoD, particularly after exposure to 10 mM VPA.

Our results are consistent with the findings of others who showed, for example, that the expression levels of osteoblastic marker genes (collagen 1*α*2, osteopontin, and osteocalcin) were significantly enhanced in the presence of 6 *μ*M VPA for one week [35]. In another study, suppression of histone deacetylase activity (using trichostatin A) early in osteoblast differentiation was shown to accelerate the differentiation process based on increased expression of osteopontin and osteocalcin by qRTPCR and calcium incorporation [34]. More recently, trichostatin A was confirmed to enhance the expression of osteogenic markers, matrix mineralization, and bone formation in murine fetal limb explants [40]. CB-derived MSC have been demonstrated to express histone deacetylase (HDAC)-1 and -2 [41]. Recent evidence suggests that specific suppression of these HDACs could have selective effects on osteoblast differentiation [42]. In addition, VPA was also reported to increase neuronal differentiation of adult neural progenitor cells and enhance osteogenic differentiation, but inhibit astrocyte, oligodendrocyte, adipocyte, and chondrocyte differentiation [37]. Various markers of neuronal differentiation, including nestin, GFAP, NeuN, and NF-M, were shown to increase in bone marrow-derived MSC pretreated with VPA [43]. Taken together, these studies demonstrate that epigenetic mechanisms play an important role in regulating the differentiation of MSC to various lineages, and HDIs like valproic acid could have additional therapeutic value.

## 5. Conclusions

Our study shows that VPA enhances the migration of CB MSC towards SDF-1 by increasing the expression of CXCR4 and CXCR7, as well as MMP-2. Short-term exposure (3 h) of MSC to a low dose of VPA (≤5 mM) could be used to increase their recruitment to sites of injury and enhance their proliferation potential while maintaining their ability to differentiate into osteocytes, chondrocytes, and myocytes. Due to the fact that it is a safe and well-tolerated drug, we suggest that VPA be used for ex vivo treatment of MSC prior to infusion not only to enhance their recruitment to injured tissues, but also to potentially support their differentiation.

## Supplementary Material

Supplementary Figure 1(A): Heterogeneity of adherent layer formed by plating cord blood (CB)-derived mononuclear cells (upper panel). At passage 4, a more homogeneous population of cells with fibroblastoid morphology is obtained (lower panel).
upplementary Figure 1(B): Immunophenotypic characterization of CB MSC. Cells showed negative expression for the hematopoietic markers CD45 and CD34 and positive expression for the stromal markers CD90, CD105 and CD73. The black lines represent isotypic controls while the colored lines and shaded area represent antigen of interest.

## Figures and Tables

**Table 1:** Primers used for gel-based RT-PCR analysis.

Primer		Sequence	Product (bp)
CXCR4	Sense:	5′-CTGCCCACCATCTACTCCAT-3′	100
	Antisense:	5′-CTTGTCCGTCATGCTTCTCA-3′	
Collagen X	Sense:	5′-AATCCCTGGACCGGCTGGAATTC-3′	267
	Antisense:	5′-TTGATGCCTGGCTGTCCTGGACC-3′	
Myogenin	Sense:	5′-TGGCCTTCCCAGATGAAACC-3′	452
	Antisense:	5′-GCATCGGGAAGAGACCAGAA-3′	
MyoD	Sense:	5′-CCTAGACTACCTGTCCAGCATC-3′	365
	Antisense:	5′-GGCGGAAACTTCAGTTCTCC-3′	
Osteocalcin	Sense:	5′-GGCAGCGAGGTAGTGAAGAGAC-3′	257
	Antisense:	5′-GGCAAGGGGAAGAGGAAAGAAG-3′	
Osterix	Sense:	5′-CTTGTGCCTGATACCTGCACT-3′	470
	Antisense:	5′-TCACTCTACCTGACCCGTCATC-3′	
CXCR7	Sense:	5′-GGCTATGACACGCACTGCTACA-3′	475
	Antisense:	5′-TGGTTGTGCTGCACGAGACT-3′	
GAPDH	Sense:	5′-CGGAGTCAACGGATTTGGTCGTAT-3′	306
	Antisense:	5′-AGCCTTCTCCATGGTTGGTGAAGAC-3′	
