# Supplementary figures and images for: Migration, Proliferation, and Differentiation of Cord Blood Mesenchymal Stromal Cells Treated with Histone Deacetylase Inhibitor Valproic Acid
Source: Stem Cells Int. 2014 Mar 16;2014:610495. doi: 10.1155/2014/610495 (PMC3976771; doi:10.1155/2014/610495)

## Slide 1
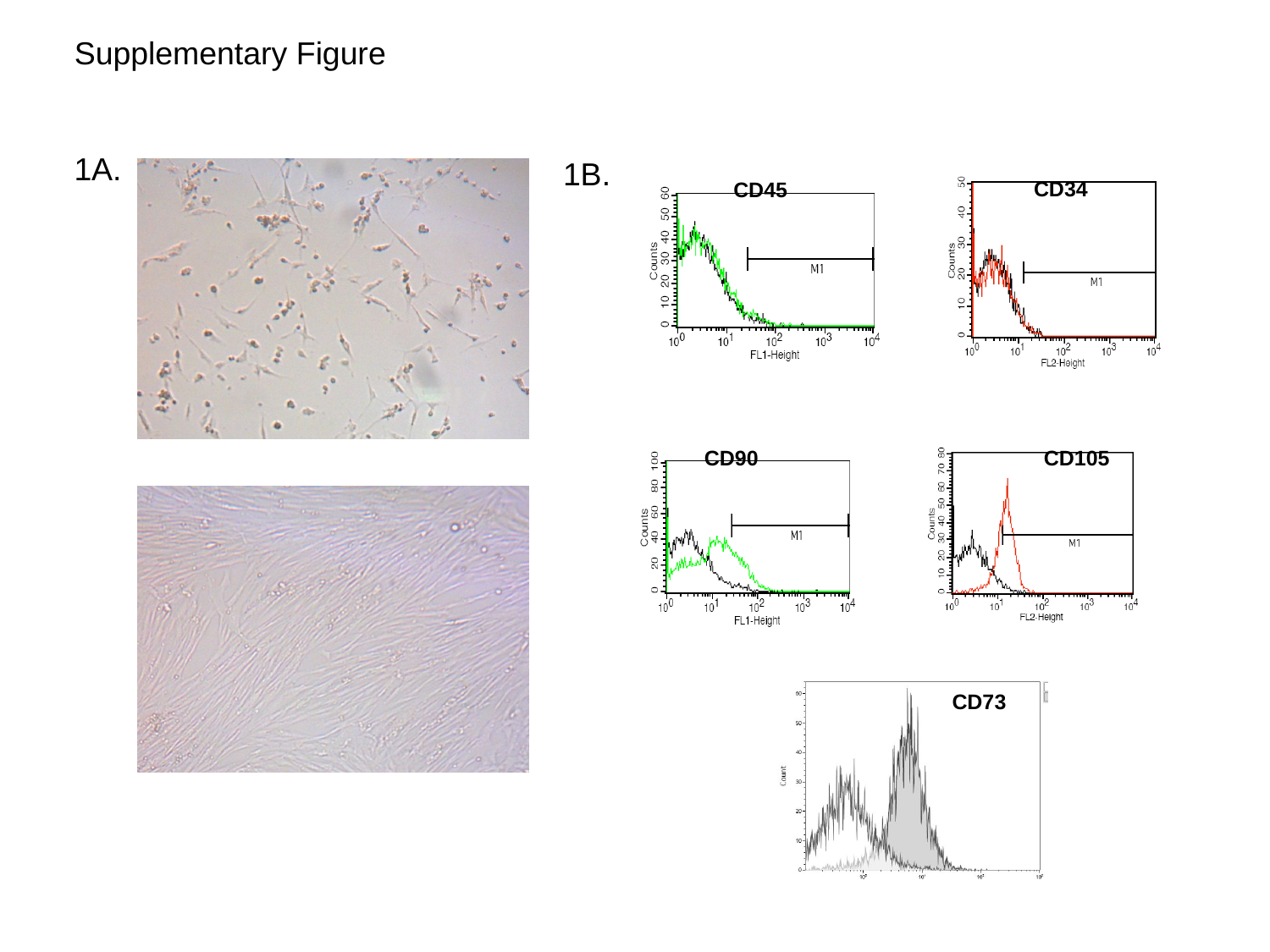

Supplementary Figure
1A.
1B.
CD34
CD45
CD90
CD105
CD73

Supplement: Supplementary file 1 — Supplementary Figure 1(A): Heterogeneity of adherent layer formed by plating cord blood (CB)-derived mononuclear cells (upper panel). At passage 4, a more homogeneous population of cells with fibroblastoid morphology is obtained (lower panel). upplementary Figure 1(B): Immunophenotypic characterization of CB MSC. Cells showed negative expression for the hematopoietic markers CD45 and CD34 and positive expression for the stromal markers CD90, CD105 and CD73. The black lines represent isotypic controls while the colored lines and shaded area represent antigen of interest. [file 610495.f1.ppt]
